# The Effect of Stacking Sequence and Ply Orientation on the Mechanical Properties of Pineapple Leaf Fibre (PALF)/Carbon Hybrid Laminate Composites

**DOI:** 10.3390/polym13030455

**Published:** 2021-01-31

**Authors:** Mohd Khairul Rabani Hashim, Mohd Shukry Abdul Majid, Mohd Ridzuan Mohd Jamir, Farizul Hafiz Kasim, Mohamed Thariq Hameed Sultan

**Affiliations:** 1 Faculty of Mechanical Engineering Technology, Universiti Malaysia Perlis (UniMAP), Perlis 02100, Malaysia; khairulrabani@unimap.edu.my (M.K.R.H.); ridzuanjamir@unimap.edu.my (M.R.M.J.); 2 Faculty of Chemical Engineering Technology, Universiti Malaysia Perlis (UniMAP), Perlis 02100, Malaysia; farizul@unimap.edu.my; 3 Frontier Materials Research, Centre of Excellence (FrontMate), Universiti Malaysia Perlis (UniMAP), Perlis 02100, Malaysia; 4 Department of Aerospace Engineering, Faculty of Engineering, Universiti Putra Malaysia, Serdang 43400, Selangor Darul Ehsan, Malaysia; 5 Aerospace Malaysia Innovation Centre (944751-A), Prime Minister’s Department, MIGHT Partnership Hub, Jalan Impact, Cyberjaya 63000, Selangor Darul Ehsan, Malaysia

**Keywords:** PALF, carbon fibre, hybrid composite, tensile properties, flexural properties

## Abstract

In this paper, the effects of stacking sequence and ply orientation on the mechanical properties of pineapple leaf fibre (PALF)/carbon hybrid laminate composites were investigated. The hybrid laminates were fabricated using a vacuum infusion technique in which the stacking sequences and ply orientations were varied, which were divided into the categories of cross-ply symmetric, angle-ply symmetric, and symmetric quasi-isotropic. The results of tensile and flexural tests showed that the laminate with interior carbon plies and ply orientation [0°, 90°] exhibited the highest tensile strength (187.67 MPa) and modulus (5.23 GPa). However, the highest flexural strength (289.46 MPa) and modulus (4.82 GPa) were recorded for the laminate with exterior carbon plies and the same ply orientation. The fracture behaviour of the laminates was determined by using scanning electron microscopy, and the results showed that failure usually initiated at the weakest PALF layer. The failure modes included fibre pull-out, fibre breaking, matrix crack, debonding, and delamination.

## 1. Introduction

In recent times, hybrid reinforcement composites have been used worldwide because of their dimensional stability and superior mechanical properties [1]. Synthetic carbon fibre is a common reinforcement material in engineering applications due to their superior capabilities; their high stiffness and modulus offer excellent toughness and firmness to bearing loads [2,3,4,5]. The main limitations of carbon reinforcement are high production costs [6,7] and resulting brittleness [8]. Moreover, depleting petroleum resources have compelled industry players to divest their attention to the recyclability and environmental sustainability of a product. Hybridisation with natural fibre minimises the overall production cost; makes the hybrid system more tolerant to damage, especially to brittle failure; and is environmentally friendly [9]. Compared with conventional synthetic fibres, natural fibres possess unique properties, such as low density, lightweight with high specific properties, low cost, and recycling options [10,11,12]. However, the mechanical, physical, and thermal properties of natural fibres are relatively low compared with their synthetic counterparts, and several measures have been taken to enhance their properties. Among the promising methods to enhance the properties of natural fibres is reinforcement by manipulating the stacking sequences and fibre orientation.

Evaluating the influence of the stacking sequence on the mechanical properties of the composite structure is imperative. Zhang et al. [13] investigated the effect of various stacking sequences on the mechanical properties of natural and glass fibre hybrid composites and found that the stacking sequence contributes to delayed composite failure by improving the interlaminar shear strength, fracture toughness, and tensile strength. Their investigation also concluded that the alternate stacking sequence is the best option, which is consistent with other studies in the literature [12,14,15]. Recently, Feng et al. reported that the mechanical properties of pineapple leaf fibre (PALF)/glass fibre hybrid composites highly depend on the fibre stacking configuration [16].

The effect of ply and fibre orientation on the mechanical properties of composite structures has been widely investigated. Krenchel [17] analysed the effect of fibre orientation on composite stiffness by the modified rule of the mixture and established the importance of composite design with respect to fibre orientation for the expected load direction. Potluri et al. [18] investigated the response of mechanical properties with various fibre orientations, namely the longitudinal, transverse, and random orientations perpendicular to the load direction. The composites with the longitudinal fibre orientation was found to deliver outstanding mechanical properties compared with the transverse and random fibre orientations, and the same result was reported by a previous study [19]. Chollakup et al. [20] reported the effect of fibre loading on the mechanical properties and stress transfer of PALF reinforced composite. Two different lengths of fibre were used in their investigation, of which the long fibre was in the longitudinal direction while the short fibre was in the random orientation. The results showed that a long fibre in the longitudinal direction effectively handles tensile load.

Commercially, pineapple fruit is considered a primary crop because they are cultivated for consumption, while the pineapple leaves are secondary crop or waste. The pineapple leaf is a precious agro-waste that is abundantly available in tropical countries, especially Malaysia. The leaf yields high cellulose fibre; the main constitutent is cellulose (70–82%) and the remaining constituents are lignin (5–12%) and ash (1.1%) [21]. Because of their excellent mechanical strength and low cost, PALF has high application potential as biodegradable plastic composites [22], reinforced polymer composites [23,24], low-density polyethylene (LDPE) composites [25], thermoset composites [23], thermoplastic composites [26], and rubber composites [27]. PALF/carbon fibre hybrid composites can be designed to meet particular applications by manipulating the matrix ratio, fibre length, stacking sequence, and fibre orientation to achieve variations in mechanical and physical properties [28,29].

Most of the past studies have only focussed on the single parameter, either fibre orientation or stacking sequence, that affects the mechanical properties of composite structures. Moreover, the tensile and flexural behaviour of PALF/carbon fibre reinforced epoxy remains unexplored. Therefore, the current investigations were carried out to explore the correlation of fibre orientation and stacking sequence on the tensile and flexural behaviour of PALF/carbon fibre hybrid composites.

## 2. Materials and Methods

### 2.1. Materials

#### 2.1.1. Raw Materials

The woven roving PALF fibre (plain weave) and woven roving carbon fiber (plain weave) were used as reinforcement. The PALF and carbon fibre were supplied by a local supplier. The epoxy polymer was ether of bisphenol A and the hardener was triethylene tetra amine; both were used as the matrix material. The weight ratio of epoxy to hardener was 100:27.8, as suggested by the manufacturer. Table 1 shows the properties of the fibres and matrix polymers used in this study.

#### 2.1.2. Alkaline Treatment

The PALF was treated for 3 h using an alkali (NaOH) solution with a concentration of 5%, and the liquor ratio was 40:1 [33]. The alkali solution was used to remove the hemicelluloses and surface impurities within the fibres. Subsequently, the PALF was repeatedly cleaned using distilled water. The PALF was then dried in an oven at 60 °C for 8 h before drying in room conditions for a further 24 h.

### 2.2. Fabrication Process

**Figure 1:**
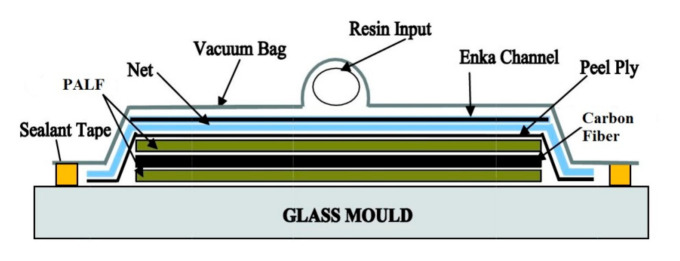
Schematic of the vacuum infusion process.

The PALF/carbon hybrid laminate composites were manufactured by a vacuum infusion process, as shown in Figure 1. The reinforcement plies were laminated over a glass mould, and the resin was infused into the lamination plies, as shown in Figure 1. During the infusion stages, the pressure in the mould was controlled to lower than 2000 Pa by using a high vacuum pump (AST 22 model AIRSPEC, Kuala Lumpur, Malaysia). The specimens were allowed to cure within the mould for 12 h at room temperature. Subsequently, post-curing was conducted at 80 °C in an oven for 2 h under air circulation [34,35]. The different layering sequences of the PALF and carbon used for the fabrication of the hybrid composites and abbreviations for the designated layering sequences are presented in Table 2.

### 2.3. Void Content

Voids in the laminates due to entrapped gasses during the manufacturing process are difficult to avoid. The voids contribute to a decrease in the mechanical properties in addition to an increase in the moisture absorption of the composites, leading to degraded physical properties. Therefore, a determination of the void content is necessary to understand and verify the mechanical properties of the laminates. The void content of the hybrid and plain PALF (treated and untreated) laminates were determined based on ASTM D2734-09. The void content (%) was calculated using Equation (1), and the theoretical density (*ρ_t_*) of the hybrid composites was determined according to Equation (2):

(1) $$V\%=\frac{\rho_{t}-\rho_{e}}{\rho_{t}}\times 100$$

(2) $$\rho_{t}=\frac{100}{\left(w_{f1}/\rho_{f1})+(w_{f2}/\rho_{f2})+(w_{p}/\rho_{p}\right)}$$

where *ρ_f_*_1_, *ρ_f_*_2_, and *ρ_p_* are the densities of the PALF, carbon fiber, and epoxy polymer, respectively; and *w_f1_, w_f2_*, and *w_p_* are the weight percentages of the PALF, carbon fiber, and epoxy polymer, respectively. The actual density of the laminates (*ρ_e_*) was determined by measuring the sample accurately using a high-accuracy electronic densimeter (MDS-300 with an accuracy of 0.0001 g/cm^3^). For each laminate, six samples with dimensions of 25 × 25 mm^2^ were considered.

### 2.4. Evaluation Method

#### 2.4.1. Tensile Strength

The laminates were subjected to tensile strength tests based on the ASTM D3039-76 standard by using a universal testing machine (Instron-3369, Instron, Norwood, MA, USA). The size of the sample was 150 × 25 × 3 ± 0.5 mm with a gauge length of 100 mm. The cross-head speed of 2 mm/min was fixed throughout the test. The tensile strength and modulus were determined for the laminates with five replicates each, and the average values were reported.

#### 2.4.2. Flexural Strength

Stability and durability under various load conditions are vital to promote the hybrid composites in various applications. Therefore, the laminates were tested with three-point bending tests according to the ASTM D7264 standard using a universal testing machine (Instron-3369, Instron, Norwood, MA, USA). The sample size was 70 × 13 × 3 ± 0.5 mm. The span length was 60 mm throughout the test, and the span-to-depth ratio was kept at 20:1. The cross-head speed was kept throughout the test at 2 mm/min. The flexural strength (*σ_F_*), modulus (*E_F_*), and strain-to-failure (*ε_F_*) were determined according to the following equations:

(3) $$\sigma_{F}=\frac{3PL}{2bd^{2}}$$

(4) $$E_{F}=\frac{mL^{3}}{4bd^{3}}$$

where *L* is the span length, *b* is the width, and *d* is the thickness of the test samples. *P* is the maximum load before failure, and *m* is the slope of the initial segment of the load versus displacement curve. The flexural strength and modulus were determined for the laminates with five replicates each, and the average values were reported.

#### 2.4.3. Scanning Electron Microscopy (SEM)

The fracture surface morphologies of the PALF/carbon hybrid laminate composites were examined using a scanning electron microscope (SEM; Hitachi TM3000, Tokyo, Japan). The fractured portions of the samples were cut, and platinum was uniformly coated over the surfaces before scanning. Scanning images were obtained at accelerating voltages of 3–5 kV under magnifications of 100× and 500×.

## 3. Results and Discussion

### 3.1. Void Contents

Table 3 shows the void contents of the PALF/carbon hybrid laminate composites. The void contents recorded for the laminates are all below the allowable limit of 5% [36]. In general, the laminates with treated PALF show slightly higher void contents than those with the untreated PALF. This is caused by the improved compatibility of PALF with matrix phase and surface conditions of the fibre after treatment. Good compatibility will directly result in good interdiffusion of both PALF and matrix. Air entrapment is mainly due to the inhomogeneous fibre architecture, resulting in non-uniform permeability of the fibre preform, which causes local variations in resin velocity [37]. This local velocity variation is exacerbated by the capillary effect, prevailing on the microscale [38]. Moreover, the laminates with ply orientation [0°, 90°]*_n_* have low void contents compared with the laminates with ply orientation [±45°]*_n_*. During infusion driven by viscous forces, the matrix resin flows first between fiber tows (macropores) and then impregnates the fiber tow (micropores) transversally. For the laminates with ply orientation [0°,90°]*_n_*, the matrix resin flow direction inline with fiber orientation 90° causes less friction [39]. A previous study by Matsuzaki et al. confirmed this trend of void formation in woven fabrics [40]. The void was formed at the flow front region of the epoxy during the infusion process and the tows lateral to the laminates [41]. Parnas et al. [42] demonstrated that tow impregnation is mainly caused by the ply at the transverse orientation rather than at parallel orientation to the flow.

### 3.2. Composite Mechanical Properties

#### 3.2.1. Tensile Properties

**Figure 2:**
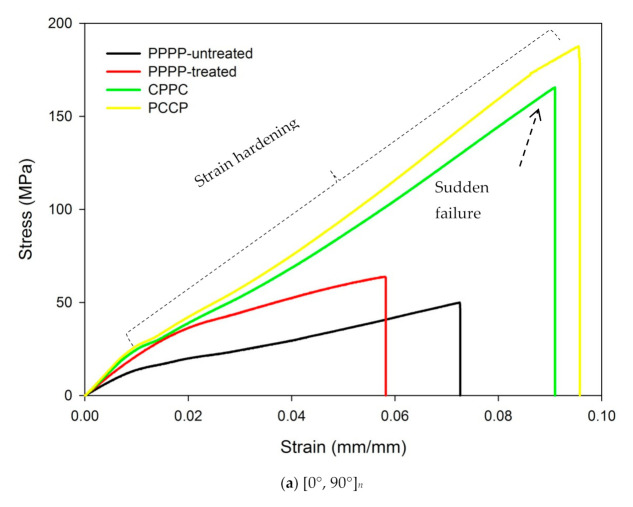

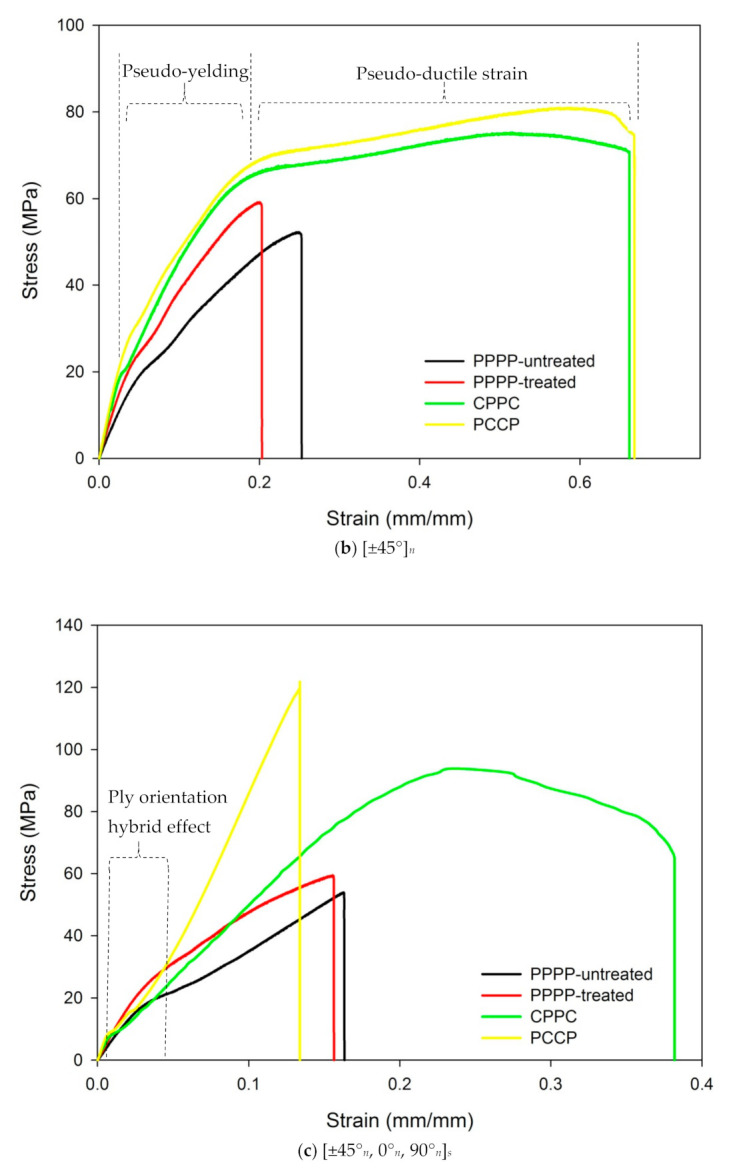

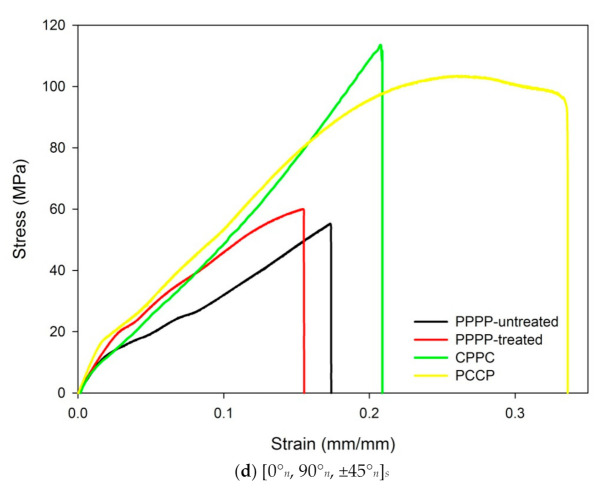
Tensile stress-strain behaviour of PALF/carbon hybrid laminate composites with different material stacking sequences: (**a**) [0°, 90°]*_n_*, (**b**) [±45°]*_n_*, (**c**) [±45°*_n_*, 0°*_n_*, 90°*_n_*]*_s_* and (**d**) [0°*_n_*, 90°*_n_*, ±45°*_n_*]*_s_*.

The tensile stress-strain curves of the PALF/carbon hybrid laminate composites obtained with different material stacking sequences are presented in Figure 2. Figure 2a shows the tensile stress-strain curves obtained for the laminates with ply orientation [0°, 90°]*_n_*. The laminate with untreated PALF (PPPP-untreated) exhibited a strength of 49.9 MPa and a strain of 0.073 mm/mm. The laminate with treated PALF (PPPP-treated) showed an increase in strength to 63.76 MPa and a decreased strain of 0.058 mm/mm. By incorporating exterior carbon layers (CPPC), the strength gradually increased by approximately 61.5% to 165.54 MPa, with a strain of 0.091 mm/mm. For the laminate with interior carbon layers (PCCP), the highest recorded strength was 187.67 MPa and the corresponding strain was 0.095 mm/mm, which was significantly different from that recorded for the laminate with the exterior carbon layers. With ply orientation [0°, 90°]*_n_*, all the laminates showed a linear elastic behaviour and failed by brittle fracture along the width of the cross section of the laminates, which was indicated by a sudden drop in stress with catastrophic failure, as shown in Figure 2a. Thus, the principal loading direction is aligned with the ply orientation, which allows the laminates to behave in an orthogonal manner, where the material behaviour has mutually perpendicular degrees of symmetry. The deformation behaviour of the laminates is influenced by the in-plane extensorial force, which behaves anisotropically [43,44].

For the laminates with ply orientation [±45°]*_n_*, as shown in Figure 2b, the laminate with untreated PALF (PPPP-untreated) exhibited a low strength of 52.25 MPa and strain of 0.265 mm/mm. The laminate with treated PALF (PPPP-treated) showed a slight increase in strength to 59.2 MPa and a decreased strain of 0.212 mm. The laminate with exterior carbon layers (CPPC) showed a slight increment in strength by approximately 21.3 to 75.25 MPa, with a strain of 0.662 mm/mm. The highest recorded strength was 80.97 MPa for the laminate with interior carbon layers (PCCP) and the corresponding strain was 0.668 mm/mm. The laminates with ply orientation [±45°]*_n_* showed a relatively higher in-plane shear stress. Ply orientation [±45°]*_n_* resulted in a high edge delamination strain [45]. This stress distribution status is consistent with the fulfilled strain distribution shown in Figure 2b, with apparent elongation of the laminates. These phenomena are related to the pseudo-ductile response of the laminates, which suppresses catastrophic delamination and the appearance of the damage modes of (i) fragmentation in the carbon ply and (ii) local delamination, before the final failure of the high-strain carbon ply [46,47].

The tensile stress-strain curves for the laminates with ply orientation [±45°*_n_*, 0°*_n_*, 90°*_n_*]*_s_* is shown in Figure 2c. The laminate with untreated PALF (PPPP-untreated) exhibited a strength of 53.94 MPa and strain of 0.188 mm/mm. The laminate with treated PALF (PPPP-treated) showed a slight increase in strength to 59.43 MPa and a decreased strain of 0.167 mm/mm. By incorporating exterior carbon layers (CPPC), the strength raised by approximately 36.6% to 93.87 MPa, with a strain of 0.38 mm/mm. For the laminate with interior carbon layers (PCCP), the highest recorded strength was 119.76 MPa with a corresponding strain of 0.25 mm/mm, which was significantly different from that recorded for the laminate with exterior carbon layers. The laminates with ply orientation [±45°*_n_*, 0°*_n_*, 90°*_n_*]*_s_* showed mixed trends in the tensile stress-strain curves, as shown in Figure 2c. This is attributable to the hybridisation effect of the ply orientation, resulting in varied stiffness along the thickness of the laminates [46]. The laminate with exterior carbon plies (CPPC) initially showed a linear-elastic behaviour and propagated toward the pseudo-yielding strain. The laminate showed a pseudo-ductile behaviour before failure. This pseudo-ductile behaviour arises from the geometric effect of the fibre reorientation as well as the yielding of the matrix that allows further non-linear strains to be taken by the laminate [48]. However, the laminates with interior carbon plies (PCCP) demonstrated a linear response in the initial stage before the yield point, followed by a gradual failure and a short plateau due to fragmentation of the interior [0°, 90°] ply. The difference is due to the presence of the [±45°] layers with different materials, where the [±45°] layers can enhance the crack propagation resistance of the laminates [49].

According to Figure 2d, the laminate with ply orientation [0°*_n_*, 90°*_n_*, ±45°*_n_*]*_s_* showed a minimum strength of 55.27 MPa and a strain of 0.174 mm/mm for untreated PALF (PPPP-untreated). The laminate with treated PALF (PPPP-treated) showed a slight increase in strength to 60 MPa and a decreased strain of 0.155 mm/mm. By incorporating interior carbon layers (PCCP), the strength gradually increased by approximately 73% to 103.5 MPa, with a strain of 0.336 mm/mm. For the laminate with exterior carbon layers (CPPC), the highest recorded strength was 113.46 MPa and the corresponding strain was 0.209 mm/mm, which was significantly different from that recorded for the laminate with interior carbon layers (PCCP). The laminates with ply orientation [0°*_n_*, 90°*_n_*, ±45°*_n_*]*_s_* showed mixed trends in the tensile stress-strain curves, as shown in Figure 2d. The laminate with interior carbon plies (PCCP) showed an initial linear-elastic behaviour and propagated towards the pseudo-yield strain. The laminate showed a pseudo-ductile behaviour before failure. However, the laminates with exterior carbon plies (CPPC) showed an initial linear response before yielding, followed by a gradual failure and a short plateau due to damages to the exterior carbon layers.

Overall, the tensile stress-strain curves of the PALF/carbon hybrid laminate composites, obtained with different material stacking sequences, are presented in Figure 2, showing a mix of trends depending on ply orientation. Comparing the laminates with ply orientations [0°, 90°]*_s_* and [±45°]*_n_*, the stress strengths were higher but the strain was lower for the laminates with ply orientation [±45°]*_n_* in all the material stacking sequences, as illustrated in Figure 2a,b. Moreover, the laminates with interior carbon plies (PCCP) showed peak tensile stress for both ply orientations. Previous studies confirmed that carbon ply contributes to the overall tensile properties of hybrid laminates [50,51]. Sezgin & Berkalp [52] reported that the adhesion between the carbon ply and the matrix is strong, and the strong bonding at the interface imparts higher tensile strength to the laminates. Furthermore, the treated PALF ply (PPPP-treated) leads to better tensile strength. The hydrophilic nature of PALF and the hydrophobic nature of the polymer matrix lead to poor interface bonding between them [53]. The alkaline treatment removes the lignin covering part of the PALF and increases the surface roughness of the fibre for better interlocking of the fibre with the matrix [54]. Chunhong et al. [55] demonstrated that alkali treatment reduces the surface polarity and exposes cellulose, thus increasing the number of possible reaction sites and contact areas between the PALF and matrix. The same trend was followed by the laminates with ply orientation [±45°*_n_*, 0°*_n_*, 90°*_n_*]*_s_*, as shown in Figure 2c. However, the laminates with ply orientation [0°*_n_*, 90°*_n_*, ±45°*_n_*]*_s_* showed a different trend as the laminates with exterior carbon layers (CPPC) have a higher load strength than those with interior carbon layers (PCCP), as shown in Figure 2d. This may be because the carbon ply orientation was not perpendicular to the axial tensile loading during the fabrication process. This finding is in good agreement with the classical laminate theory, which has been discussed in previous studies [56,57]. Based on the tensile stress-strain curves of the PALF/carbon hybrid laminate composite, the highest tensile stress was obtained for ply orientation [0°, 90°]*_n_*, while the highest tensile strain was recorded for ply orientation [±45°]*_n_*. A similar observation was reported in previous studies [58,59,60]. There is a significant effect of ply orientation and material stacking sequence on the tensile properties of the laminates. The tensile strength of the laminate with interior carbon layers (PCCP) and ply orientation [0°, 90°]*_n_* (187.67 MPa) was only 13% greater than that of the laminate with exterior carbon layers (CPPC) and the same ply orientation (165.54 MPa). The tensile strengths of the PALF/carbon laminates with ply orientations [0°, 90°]*_n_* and [±45°]*_n_* were 187.67 MPa and 80.97 MPa, respectively, yielding a difference of 130%. Thus, the material stacking sequence has a smaller effect than the ply orientation on the tensile strength of the laminate.

**Figure 3:**
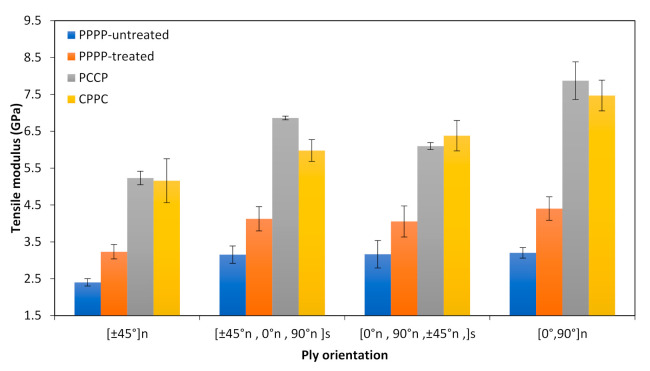
Tensile modulus of PALF/carbon hybrid laminate composites with different ply orientations.

The results for the tensile modulus of the PALF/carbon hybrid laminate composites with different ply orientations are shown in Figure 3. The laminates with ply orientation [±45°]*_n_* showed the lowest tensile modulus for the different material stacking sequences, which were 2.40, 3.23, 5.23, and 5.16 GPa for the PPPP-untreated PALF, PPPP-treated PALF, PCCP, and CPPC laminates, respectively. The highest tensile modulus for the different material stacking sequences were recorded for the laminates with ply orientation [0°, 90°]*_n_*, which were 3.20, 4.40, 7.87, and 7.47 GPa for the PPPP-untreated PALF, PPPP-treated PALF, PCCP, and CPPC laminates, respectively. Evidently, ply orientation influences the tensile modulus of the laminates. With ply orientation [±45°*_n_*, 0°*_n_*, 90°*_n_*]*_s_*, the tensile modulus of the PPPP-untreated and PPPP-treated laminates were 3.15 and 4.12 GPa, respectively, while that of the PCCP and CPPC laminates were 6.86 and 5.98 GPa, respectively, indicating that the material stacking sequence also influences the tensile modulus of the laminates. This trend was followed by the laminates with ply orientation [0°*_n_*, 90°*_n_*, ±45°*_n_*]_s_, with tensile moduli of 3.17, 4.05, 6.09, and 6.38 GPa for the PPPP-untreated PALF, PPPP-treated PALF, PCCP, and CPPC laminates, respectively. Overall, fibre treatment affected the tensile modulus more than it did the tensile strength, as presented in Figure 2 and Figure 3. A similar finding was reported by previous studies [21,61,62]. This situation occurred because the interface bonding between the fibre was improved by fibre treatment, causing the composite to exhibit ductile behaviour [63]. Moreover, the tensile modulus of the laminates was influenced by the positioning of the carbon ply. This finding is consistent with past studies in which the overall tensile performance highly depends on the strength of the core or interior fibre in the laminate structure [64,65]. Another study by Sezgin & Berkalp [52] established the same finding with the theory that adhesion interface bonding between the matrix is promising when the carbon fibre is present in the core or interior.

#### 3.2.2. Flexural Properties

**Figure 4:**
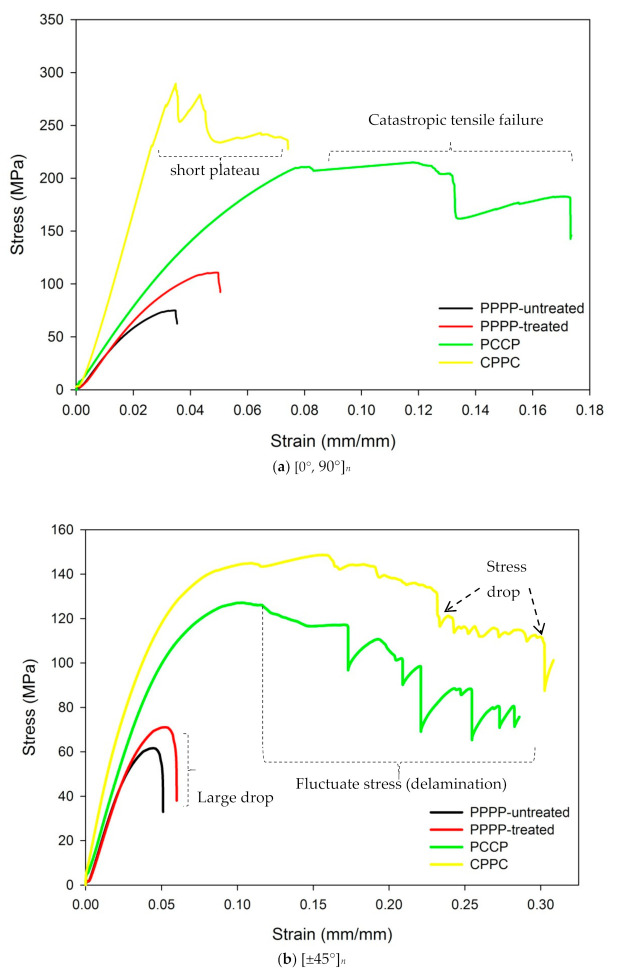

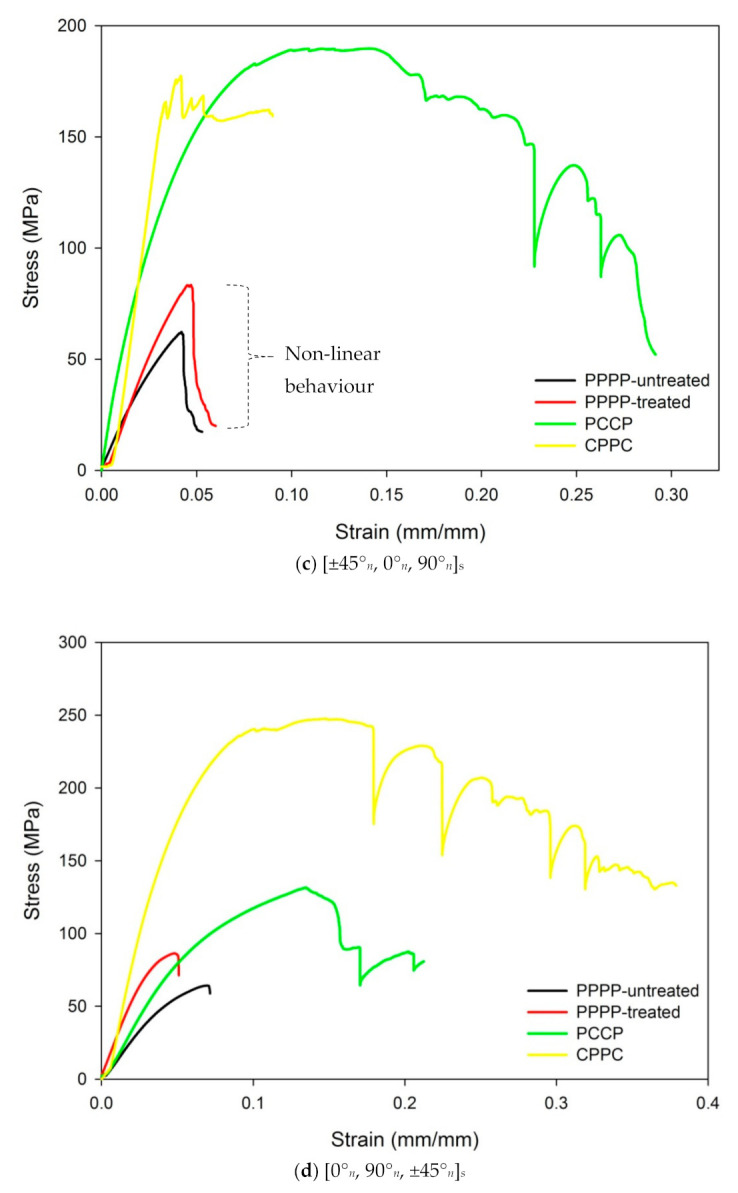
Flexural stress-strain behaviour of PALF/carbon hybrid laminate composites with different material stacking sequences: (**a**) [0°, 90°]*_n_*, (**b**) [±45°]*_n_*, (**c**) [±45°*_n_*, 0°*_n_*, 90°*_n_*]*_s_* and (**d**) [0°*_n_*, 90°*_n_*, ±45°*_n_*]*_s_*.

Figure 4 shows the stress-strain curves of the PALF/carbon hybrid laminate composites subjected to flexural testing. From Figure 4a, the laminates with ply orientation [0°, 90°]*_n_* failed at average flexural stresses of 62.44, 110.97, 228.26, and 289.46 MPa for the PPPP-untreated PALF, PPPP-treated PALF, PCCP, and CPPC laminates, respectively. The laminates showed typical linearly elastic flexural behaviour until reaching a critical point without noticeable damage. At that stage, cracks started to nucleate and grow on the compressive side on the laminates, causing the load curve to deviate slowly from the linear response [66]. The laminates with untreated (PPPP-untreated) and treated PALF (PPPP-treated) showed sudden failure, indicating the brittleness of the laminates [67]. The laminate with exterior carbon plies (CPPC) showed a drop in stress corresponding with the crack growth from the compressive side of the exterior carbon ply, which propagated through the thickness of the laminate. This phenomenon indicated by the very short plateau led to the inevitable collapse of the laminate in which some cracks bent around the carbon/PALF ply interface. For the laminate with interior carbon plies (PCCP), the exterior PALF ply was not able to bend the crack because the stresses were too high at that distance from the neutral axis; therefore, the damage propagated through the PALF ply side to overcome its tensile strength, consequently causing catastrophic tensile failure. These results are consistent with the studies by Zhang et. al. and Pinto et al. on hybrid carbon/glass and carbon/hemp laminates, respectively [6,66].

Figure 4b shows the flexural response of the laminates with ply orientation [±45°]*_n_*. The untreated PALF (PPPP-untreated) laminate failed at an average flexural stress of 61.63 MPa, while the treated PALF (PPPP-treated) laminate yielded at a slightly increased stress of 67.96 MPa. The laminate with interior carbon plies (PCCP) showed a greater flexural stress than the treated PALF (PPPP-treated) laminate; at 121.68 MPa, an improvement of up to 79% was achieved. The highest flexural stress of 158.69 MPa, though, was recorded for the laminate with exterior carbon plies (CPPC). In general, the laminates showed linear elastic behaviour at the beginning until they reached the critical point. The laminates with untreated (PPPP-untreated) and treated PALF (PPPP-treated) exhibited a massive drop in stress before a failure occurred. The laminates showed similar brittle behaviour, with matrix cracking initiated on the compressive side along the fibre direction. The resin crack, once initiated at the maximum load, moved catastrophically through the laminates. The laminates with exterior and interior carbon plies (CPPC & PCCP) showed mixed flexural responses due to the interaction between the two different materials (PALF and carbon) caused by a modification in the failure mode. For the laminate with exterior carbon plies (CPPC), a large drop in the stress-strain curves occurred after the laminate reached the critical point. The cracking damage propagated from the exterior PALF ply to the interior carbon ply. Delamination between the PALF and carbon ply caused the load to increase slightly until it reached the second critical point. The responses recurred until the sudden failure of the entire laminate. This events suggested the existence of different failure and damage propagation mechanisms, which were caused by the mismatch in mechanical properties between the carbon and PALF ply [66]. The laminate with interior carbon plies (PCCP) showed only two large stress drops due to the weak in-plane layer of the interior PALF ply. The cracking propagated from the exterior carbon ply to the interior PALF ply, which caused only two clear large stress drops in the curve triggered by the compressive and tensile side of the carbon ply. Delamination did not occur between the carbon and PALF ply. The laminates with PCCP and CPPC showed pseudo-ductile behaviour due to the shearing effect of the 45° ply [68,69]. High strains to failure were observed under flexural and tensile loading.

The flexural stress-strain curves of the laminates with ply orientation [±45°*_n_*, 0°*_n_*, 90°*_n_*]*_s_* are shown in Figure 4c. The laminates with untreated and treated PALF (PPPP-untreated & PPPP-treated) exhibited strengths of 62.11 and 83.36 MPa, respectively. The flexural stress markedly increased for the laminates with interior and exterior carbon ply (PCCP & CPPC) stacking sequences: 189.84 and 177.45 MPa, respectively. The laminates with untreated and treated PALF (PPPP-untreated & PPPP-treated) showed similar non-linear stress-strain responses and high strains to failure were observed, leading to a pseudo-ductile performance. In this case, the load increased very slowly until it moderately decreased after the maximum load. The laminate failed primarily due to matrix cracking and delamination as a result of high free-edge interlaminar stresses on the compressive side, which propagated through the specimen until it reached the tensile side [50]. The laminate with interior carbon plies (PCCP) displayed the delamination failure mode due to the local shear effect. Delamination may give rise to small load drops on the stress-strain curves. The laminate with exterior carbon plies (CPPC) showed a short plateau failure after reaching the critical point.

According to Figure 4d, the laminates with ply orientation [0°*_n_*, 90°*_n_*, ±45°*_n_*]*_s_* showed mixed responses. The laminate with untreated PALF (PPPP-untreated) exhibited failure at an average flexural stresses of 64.19 MPa. The laminate with treated PALF (PPPP-treated) showed a slight increase in strength to 86.37 MPa. By incorporating interior carbon layers (PCCP), the strength gradually doubled to 131.57 MPa. For the laminate with exterior carbon layers (CPPC), the highest recorded strength was 247.61 MPa, which was significantly different from that recorded for the laminate with interior carbon layers. The laminates with untreated and treated PALF (PPPP-untreated & PPPP-treated) showed typical brittle behaviour. The laminates with interior and exterior carbon plies (PCCP and CPPC) showed mixed flexural responses. Delamination and fibre/matrix interfacial fracture occurred in the 90°, and ±45° layers; consequently, fibre breakage of the 0° layers occurred on the compressive side at an early stage. This may be due to the predominance of the compressive mode of failure on the properties of the laminate [70].

**Figure 5:**
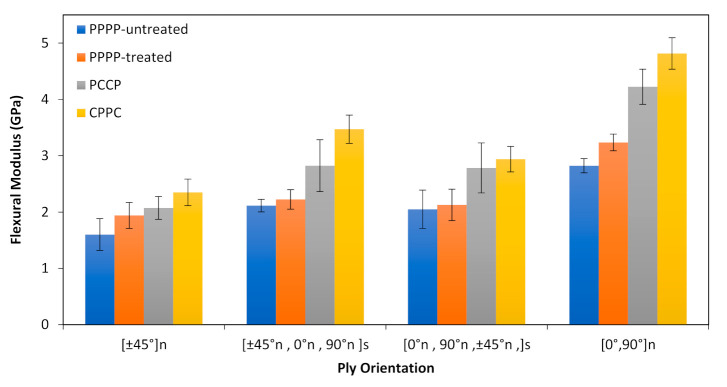
Flexural modulus of PALF/carbon hybrid laminate composites with different ply orientations.

The results for the flexural modulus of the PALF/carbon hybrid laminate composites with different ply orientations are shown in Figure 5. The laminates with ply orientation [±45°]*_n_* showed the lowest flexural modulus for different material stacking sequences: 1.6, 1.93, 2.07, and 2.35 GPa for the PPPP-untreated, PPPP-treated PALF, PCCP, and CPPC laminates, respectively. The highest flexural moduli for different material stacking sequences were recorded for the laminates with ply orientation [0°, 90°]*_n_*: 2.82, 3.24, 4.22, and 4.82 GPa for the PPPP-untreated, PPPP-treated PALF, PCCP, and CPPC laminates, respectively. Evidently, ply orientation influences the flexural modulus of the laminates. For ply orientation [±45°*_n_*, 0°*_n_*, 90°*_n_*]*_s_*, the flexural moduli of the untreated and treated PALF laminates (PPPP-untreated & PPPP-treated) were respectively 2.11 and 2.22 GPa and that of the laminates with interior and exterior carbon layers (PCCP and CPPC) were respectively 2.82 and 3.47 GPa. Thus, the material stacking sequence also affects the flexural modulus. This trend is followed by the laminates with ply orientation [0°*_n_*, 90°*_n_*, ±45°*_n_*]*_s_*, with flexural moduli of 2.05, 2.13, 2.78, and 2.94 GPa for the PPPP-untreated, PPPP-treated PALF, PCCP, and CPPC laminates, respectively. Overall, the flexural modulus of the quasi-isotropic laminate, [±45°*_n_*, 0°*_n_*, 90°*_n_*]*_s_* and [0°*_n_*, 90°*_n_*, ±45°*_n_*]*_s_*, was in between those of the cross-ply and angle-ply laminates. The laminate with ply orientation [0°, 90°]*_n_* showed the best fibre orientation with an improvement between 75% and 104% from the [±45°]*_n_* ply orientation. This finding is consistent with the findings of past studies by Caminero et al., which suggested the same trend for the carbon/epoxy laminate [50]. This occurrence can be explained by off-axis effects, such as off-axis matrix cracking, which can degrade the laminate stiffness [68,71,72].

### 3.3. Surface Fracture Morphology

#### 3.3.1. Tensile Fracture

**Figure 6:**
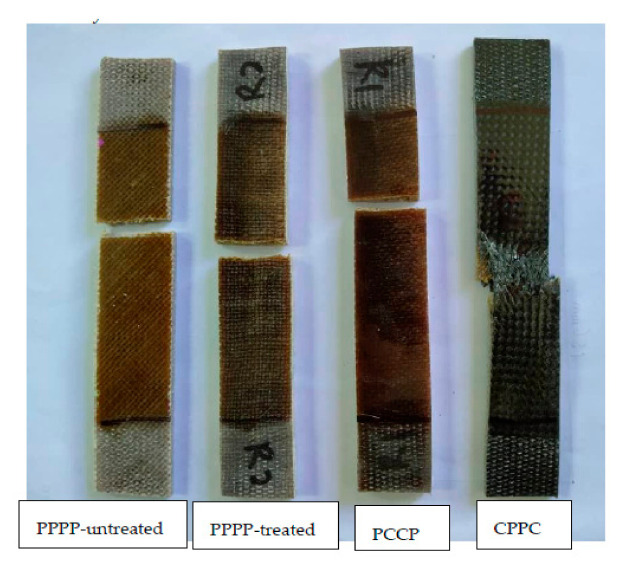
Fracture after tensile test for laminates with ply orientations [±45°, 0°, 90°].

**Figure 7:**
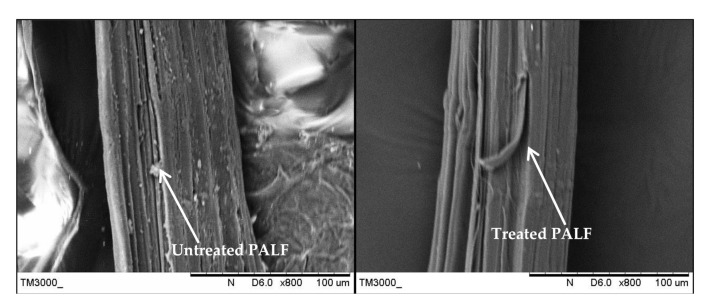
Scanning electron microscopy images of the untreated and treated PALF fibers.

**Figure 8:**
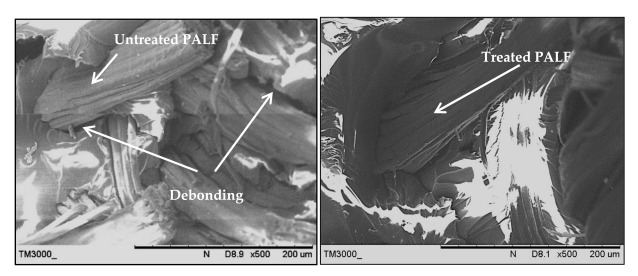
Scanning electron microscopy images of the untreated PALF laminate (PPPP-untreated) and the treated PALF laminate (PPPP-treated) with ply orientation [±45°]*_n_*.

**Figure 9:**
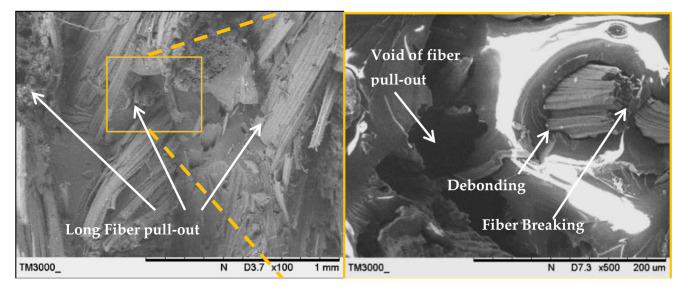
Scanning electron microscopy images of the untreated PALF laminate (PPPP-untreated) with ply orientation [±45°]*_n_* at 100 and 500 magnifications.

**Figure 10:**
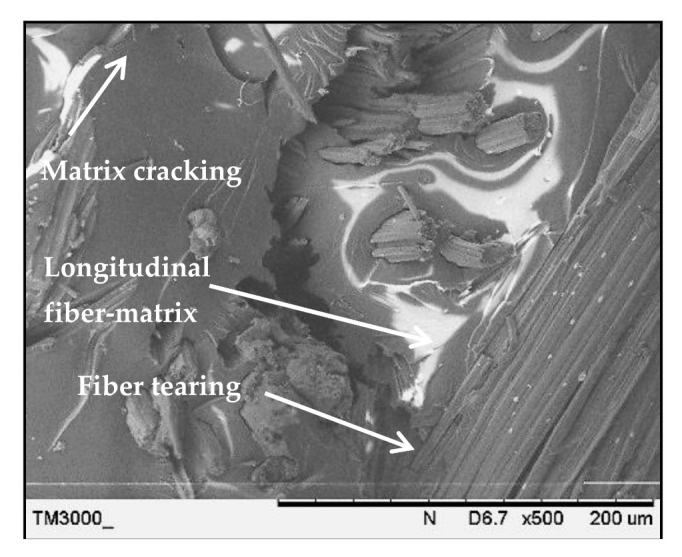
Scanning electron microscopy images of the treated PALF laminate (PPPP-treated) with ply orientation [0°, 90°].

**Figure 11:**
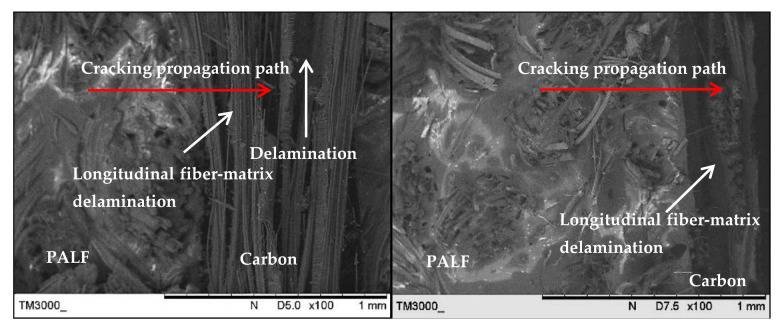
Scanning electron microscopy images of the laminate with a carbon ply interior (PCCP) and ply orientation [±45°*_n_*, 0°*_n_*, 90°*_n_*]*_s_*and the laminate with a carbon ply exterior (CPPC) and ply orientation [0°*_n_*, 90°*_n_*, ±45°*_n_*]*_s_*.

The fracture surface morphologies of the PALF/carbon hybrid laminate composites after tensile rupture are shown in Figure 6, Figure 7, Figure 8, Figure 9, Figure 10 and Figure 11. Figure 6 shows images of fracture after tensile for laminates with ply orientations [±45°,0°, 90°]. Scanning electron microscopy images of the untreated and treated PALF fibers are shown in Figure 7. The surface impurities was removed by the treatment, observing that treated PALF is cleaner than untreated PALF. The elimination of the impurities promote a reduction in the mass and an improvement in the surface area of the PALF [73]. Figure 8 shows the images of the untreated PALF laminate (PPPP-untreated) and the treated PALF laminate (PPPP-treated) with ply orientation [±45°]*_n_*. From the images, debonding failure was identified between the interface of the untreated PALF and the epoxy matrix, indicating poor interfacial bonding between the untreated PALF and the epoxy matrix due to the hydrophilic nature of the untreated PALF and the hydrophobic nature of the epoxy matrix. By contrast, for the treated PALF composites, the alkaline treatment removed the waxy layers and impurities on the PALF surface, resulting in fibrillation of the fibres; fibrillation enlarged the effective surface area between the fibre and matrix and thus enhanced the interlocking between them.

Figure 9 shows an image of the untreated PALF laminate (PPPP-untreated) with ply orientation [±45°]*_n_*; the surface morphology was rough and exhibited typical long fibre pull-out and fibre breaking. The pull-out fracture is dominated by frictional shear stress acting over the debonded interface during debonding and after delamination is completed. The pull-out mechanism contributes to the energy dissipation capacity of the laminate as friction at the interface [74]. The formation of voids by fibre pull-out indicates the low toughness of the laminate [75]. Short and long fibre pull-out fractures occurred for laminates with ply orientations [0°, 90°] and [±45°], respectively. Laminate failure under short fibre pull-out indicates brittleness, while long fibre pull-out points to pseudo-ductility.

Figure 10 shows the SEM image of the treated PALF laminate (PPPP-treated) with ply orientation [0°, 90°]. Matrix cracking was detected on the laminate fracture surface where it developed at the free edges of a test laminate and this stressed state changed with distance from the edge, propagating across the laminate thickness for the cross-ply laminate [76]. Crack formation contributes to local stress redistribution to reduce the laminate stiffness [77]. Moreover, the image shows longitudinal fibre-matrix delamination along the laminate width, which is the failure caused by delamination of the fibre at orientation 0° after experiencing perpendicular axial loading. Fibre tearing was also observed in abundance in the longitudinal fibre matrix, indicating strong interfacial bonding between the fibre and matrix.

Figure 11 shows the SEM images of the laminate with a carbon ply interior (PCCP) and ply orientation [±45°*_n_*, 0°*_n_*, 90°*_n_*]*_s_* and the laminate with exterior carbon plies (CPPC) and ply orientation [0°*_n_*, 90°*_n_*, ±45°*_n_*]*_s_*. Both laminates showed mix fracture behaviour modes caused by the interaction of two different layering techniques. The image of the laminate with interior carbon plies (PCCP) showed that failure was initiated at the exterior PALF layer and propagated to the interior carbon layer, which was supported by the long fibre pull-out of the PALF layer. Delamination was also observed in the region between the carbon/PALF interface and carbon/carbon interface, demonstrating better ductility of the carbon layers. For the laminate with exterior carbon layers (CPPC), the cracking at the early stages of damage of the matrix and the carbon fiber was blunted at the PALF/matrix interface to decelerate the damage propagation until the PALF failed primarily from debonding and fibre pull-out. This was attributable to the brittle failure behaviour of the laminate. Based on both failure modes, failure of the laminates was initiated from the exterior to the interior layer. Longitudinal fibre-matrix delamination fracture was identified for the 0°/90° layer.

#### 3.3.2. Flexural Fracture

**Figure 12:**
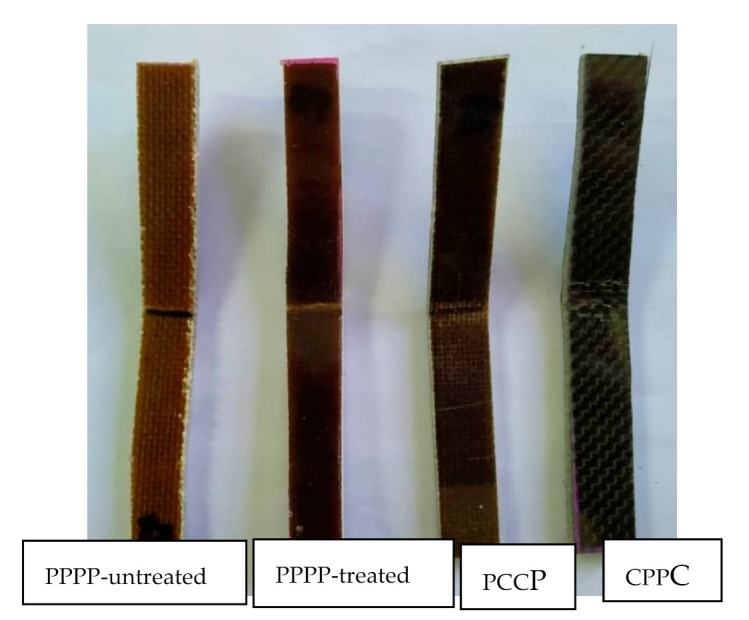
Fracture after tensile test for laminates with ply orientations [0°, 90°, ±45°].

**Figure 13:**
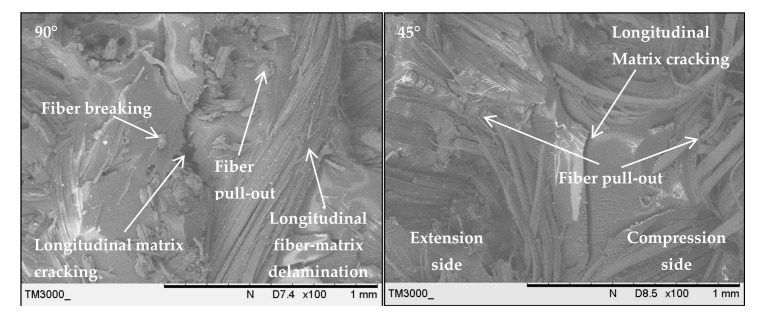
Scanning electron microscopy images of the treated PALF laminate (PPPP-treated) with ply orientations [0°, 90°] and [±45°].

**Figure 14:**
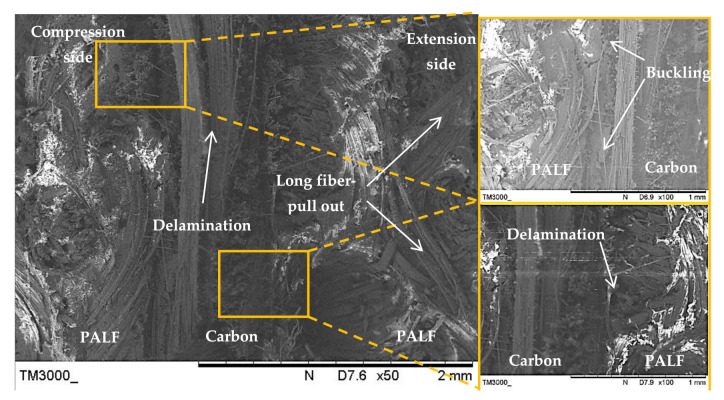
Scanning electron microscope images of the laminate with a carbon ply interior (PCCP) and ply orientation [0°, 90°].

**Figure 15:**
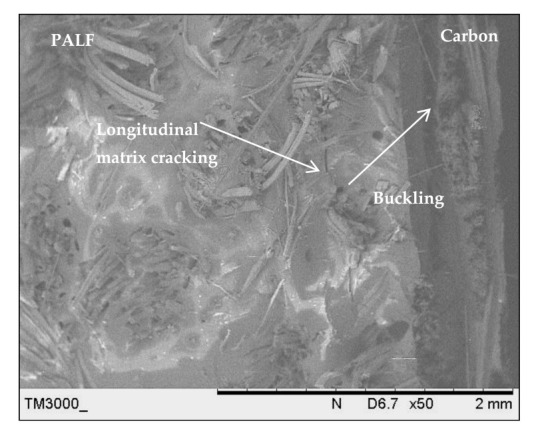
Scanning electron microscopy image of the laminate with a carbon ply exterior (CPPC) and ply orientation [0°, 90°].

The fracture surface morphologies of the PALF/carbon hybrid laminate composites after flexural rupture are shown in Figure 12, Figure 13, Figure 14 and Figure 15. Figure 12 shows examples of fracture after flexural for laminates with ply orientations [0°, 90°, ±45°]. Figure 13 shows the SEM images of the treated PALF laminate (PPPP-treated) with ply orientations [0°, 90°] and [±45°]. The instabilities of the laminates under flexural loading contributed to longitudinal matrix cracking [78], which can be seen in the images. The laminate with ply orientation [0°, 90°] exhibited fibre pull-out (for 0° fibre orientation) and longitudinal fibre-matrix delamination (for 90° fibre orientation) on the compression side, while for the tension side showed fibre pull-out. The laminate with ply orientation [±45°] showed the same fracture mode for the extension and compression sides, namely the fibre pull-out fracture. The length of fibre pull-out for the extension side was found to be longer than that observed from the compression side.

Figure 14 shows the SEM image of the laminate with a carbon ply interior (PCCP) and ply orientation [0°, 90°]. Evidently, cracking was initiated from the exterior PALF ply on the tension side, which was supported by the long fibre-pull out facture. The cracking was blunted at the PALF/carbon interface, causing delamination failure. The cracking energy was transferred to the exterior PALF ply at the compression side, causing delamination at the PALF/carbon interface without damaging the carbon fibre because of the superior properties of the carbon fibre. The load intensity slowly developed until the occurrence of primary failure from carbon fibre breaking and delamination.

Figure 15 shows the SEM image of the laminate with a carbon ply exterior (CPPC) and ply orientation [0°, 90°]. The cracks propagated rapidly in the carbon layers and buckled at the PALF layers. The exterior of the carbon layer on the compression side absorbed most of the applied load [67]. This resulted in excellent tensile strength and modulus of the laminate. Delamination was also observed in the region between the carbon and PALF at the extension side.

## 4. Conclusions

The effects of stacking sequence and ply orientation on the mechanical properties of PALF/carbon hybrid laminate composites were investigated. The laminates were fabricated with different configurations of stacking sequence and ply orientation. The laminates were prepared for tensile tests, flexural tests, and morphological observations. Based on the results of the study, the following conclusions can be drawn:

1. The maximum tensile strength and modulus were recorded for the laminate with interior carbon plies (PCCP) and ply orientation [0°, 90°]. This is because the principal loading direction was aligned with the ply orientation, thus allowing the laminates to behave in an orthogonal manner, where the material had mutually perpendicular degrees of symmetry. The carbon ply interior (PCCP) contributed to the overall tensile properties of the laminate because the adhesion between the carbon ply and matrix was strong; this strong bonding at the interface imparted higher tensile strength to the laminates.
2. When the laminates were tested in the flexural mode, the highest flexural strength and modulus were identified for the laminate with exterior carbon plies (CPPC) and ply orientation [0°, 90°]. The exterior carbon layer on the compression side absorbed most of the applied load.
3. Morphological analyses indicated that the laminates failed under tensile and flexural loading in typical modes of fibre pull-out, fibre breaking, delamination, debonding, and matrix cracking. For tensile loading, cracking propagated from the exterior layer to the interior layer. For flexural loading, failure initiated at the exterior extension side before slowly developing to the compression side.

## Figures and Tables

**Table 1:** Material properties fibres and matrix polymers.

Property	PALF	Carbon Fibre	Epoxy
Tensile Strength (MPa)	148.44	3530	55
Tensile Modulus (GPa)	10.46	230	1.75
Strain of failure (%)	1.05	1.5	6
Reference	[30]	[31]	[32]

**Table 2:** Ply orientation and stacking sequence of the composites: PALF (P) and carbon fibre (C).

Lamination	Orientation	Layering Pattern	Fibre Volume Fraction (%)		
			PALF	Carbon Fiber	Total
Cross-ply symmetric	[0°, 90°]*_n_*	PPPP-untreated	24	-	24
		PPPP-treated	21	-	21
		PCCP	16.7	6.2	22.9
		CPPC	16.7	6.2	22.9
Angle-ply symmetric	[±45°]*_n_*	PPPP-untreated	24	-	24
		PPPP-treated	21	-	21
		PCCP	16.7	6.2	22.9
		CPPC	16.7	6.2	22.9
Symmetric Quasi-isotropic	[±45°*_n_*, 0°*_n_*, 90°*_n_*]*_s_*	PPPP-untreated	24	-	24
		PPPP-treated	21	-	21
		PCCP	16.7	6.2	22.9
		CPPC	16.7	6.2	22.9
	[0°*_n_*, 90°*_n_*, ±45°*_n_*]*_s_*	PPPP-untreated	24	-	24
		PPPP-treated	21	-	21
		PCCP	16.7	6.2	22.9
		CPPC	16.7	6.2	22.9

**Table 3:** Void contents of the laminates.

Orientation	Layering	Void Content (%)
[0°, 90°]*_n_*	PPPP-untreated	1.63 ± 0.03
	PPPP-treated	2.33 ± 0.02
	PCCP	0.99 ± 0.14
	CPPC	1.23 ± 0.04
[±45°]*_n_*	PPPP-untreated	2.69 ± 0.10
	PPPP-treated	2.99 ± 0.16
	PCCP	2.33 ± 0.04
	CPPC	2.02 ± 0.05
[±45°*_n_*, 0°*_n_*, 90°*_n_*]*_s_*	PPPP-untreated	1.73 ± 0.04
	PPPP-treated	2.63 ± 0.06
	PCCP	2.02 ± 0.07
	CPPC	2.23 ± 0.11
[0°*_n_*, 90°*_n_*, ±45°*_n_*]*_s_*	PPPP-untreated	1.86 ± 0.06
	PPPP-treated	2.53 ± 0.09
	PCCP	2.03 ± 0.09
	CPPC	1.53 ± 0.04

## Data Availability

There are no linked research datasets for this submission. Data will be made available on request.
